# Understanding the Nano–Bio Interactions and the Corresponding Biological Responses

**DOI:** 10.3389/fchem.2020.00446

**Published:** 2020-06-10

**Authors:** Xin Tian, Yu Chong, Cuicui Ge

**Affiliations:** State Key Laboratory of Radiation Medicine and Protection, School of Radiation Medicine and Protection, School for Radiological and Interdisciplinary Sciences (RAD-X), Collaborative Innovation Center of Radiation Medicine of Jiangsu Higher Education Institutions, Soochow University, Suzhou, China

**Keywords:** nanomaterials, nano–bio interface, corona, reactive oxygen species, nanomedicine

## Abstract

Due to the increasing amount of work being put into the development of nanotechnology, the field of nanomaterials holds great promise for revolutionizing biomedicine. However, insufficient understanding of nanomaterial-biological microenvironment (nano−bio) interactions hinders the clinical translation of nanomedicine. Therefore, a systematic understanding of nano−bio interaction is needed for the intelligent design of safe and effective nanomaterials for biomedical applications. In this review, we summarize the latest experimental and theoretical developments in the fields of nano−bio interfaces and corresponding biological outcomes from the perspective of corona and redox reactions. We also show that nano–bio interaction can offer a variety of multifunctional platforms with a broad range of applications in the field of biomedicine. The potential challenges and opportunities in the study of nano–bio interfaces are also provided.

## Introduction

In recent years, the use of nanomaterials for targeted delivering and controlled releasing drugs, crossing biological barriers, activating immune cells, and reacting with redox species for diseases treatment (Zhang et al., 2018; Cai and Chen, 2019; Liu et al., 2019; Yang et al., 2019; Zhao et al., 2019) has been widely investigated. However, insufficient understanding of the interactions of nanomaterials with biological molecules and structures (such as, proteins, membranes, phospholipids, DNA, and free radicals) hinders the application of nanomedicine (Tian et al., 2016; Fang et al., 2017; Li et al., 2018; Yu et al., 2019). Upon entering into biological fluids, engineered nanomaterials can rapidly interact with various biomolecules, which mainly contain the three following aspects: (1) adsorption of biomolecules on the surface of nanomaterials, forming protein corona; (2) reconstruction and change of functional proteins; and (3) redox reactions between nanomaterials and reactive species (Scheme S1). These nano–bio interactions will not only greatly influence the function and fate of nanomaterials, but also affect cellular biological function (Liu et al., 2013). Therefore, it is important to evaluate the basic mechanisms of the reactions at nanomaterial–biology (nano–bio) interfaces and find strategies to manipulate the nano–bio reactions.

In this review, we reviewed current literature on the basic understanding of nano–bio interactions and their biological outcomes from associated nanotoxicity to promising biomedicine development including neurological disorders, bacterial infection, and cancer therapy. The knowledge presented here could lead to a better understanding of the nano–bio reactions and bring benefits to the development of nanomedicine.

## Nanoparticle-Protein Interaction at the Nano-Bio Interface

**Figure 1:**
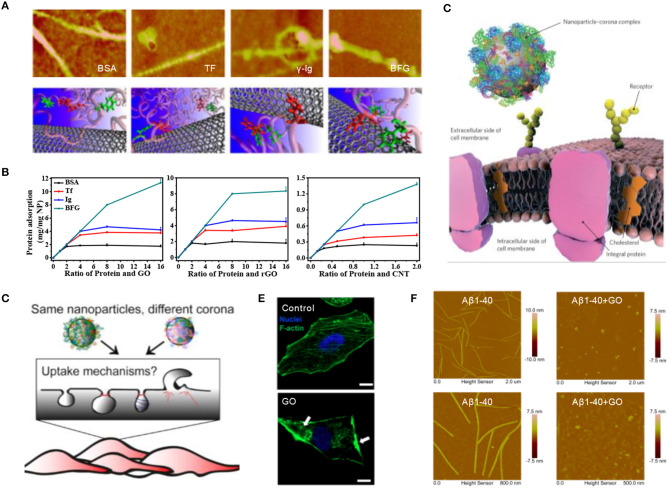
Nanoparticle-protein interaction at the nano-bio interface. **(A)** AFM images and molecular modeling illustrations of SWCNTs coated by four abundant blood proteins (Ge et al., 2011) (with permission of National Academy of Sciences of the United States of America). **(B)** Quantitative analysis of serum proteins adsorption onto various carbon-based nanoparticles including GO, rGO, and SWCNTs (Chong et al., 2015) (with permission of American Chemical Society). **(C)** The interaction of nanoparticle–corona complex, rather than the bare nanoparticle, with biological machinery (Monopoli et al., 2012) (with permission of The Royal Society of Chemistry). **(D)** The recognition of protein coronas with diverse composition by different cell receptors, leading to the internalization *via* different mechanisms. (Francia et al., 2019) (with permission of Springer Nature). **(E)** Disruption of GO nanosheets on the actin cytoskeleton of A549 cells (Tian et al., 2017) (with permission of WILEY-VCH Verlag GmbH & Co. KGaA.). **(F)** AFM images of Aβ1-40 fibrils dissociated by GO treatment (Yang et al., 2015) (with permission of The Royal Society of Chemistry).

Once entering the biological environment, engineered nanomaterials will immediately interact with surrounding biomolecules, especially the most abundant proteins, resulting in the formation of so-called protein corona, a term first coined by Dawson and co-workers in 2007 (Cedervall et al., 2007). Subsequently, we investigated the competitive binding of single-wall carbon nanotubes (SWCNTs) with highly abundant blood proteins [i.e., BSA, transferrin (Tf), gamma globulin (Ig), and bovine fibrinogen (BFG)] and found that hydrophobic interactions, especially π-π stacking interactions, are the driving forces behind the strong adsorption of serum proteins (Figure 1A) (Ge et al., 2011). Interestingly, two-dimensional graphene oxide (GO) nanosheets showed a much higher protein adsorption capacity than one-dimensional SWCNTs, although they exhibited similar binding model features (Figure 1B) (Chong et al., 2015). In addition, the nanoparticle-protein interaction underwent an intrinsically dynamic exchange at the nano-bio interface, forming “hard corona” containing higher affinity proteins or “soft corona” composed of lower affinity proteins (Chen et al., 2016).

The presence of protein corona could affect the internalization and biodistribution of nanoparticles, even altering immune system activation and the final fate of nanomaterials (Figure 1C) (Monopoli et al., 2012). For instance, BSA corona greatly decreased the cellular uptake of GO by limiting its penetration into the cell membrane via the reduction of available surface area and lipid bilayer damaging (Duan et al., 2015). Corona's composition induced different endocytic pathways since the surface of nanoparticles was recognized by diverse cell receptors (Figure 1D) (Francia et al., 2019). When nanomaterials meet cell-conditioned media, such as, immune cells, it was found that the secretion of several specific cytokines could be either increased or mitigated owing to the formation of different protein corona on the surface of nanomaterials (Dai et al., 2017). In addition, upon the addition of graphene nanosheets, the structure of the intracellular cytoskeleton was dramatically disrupted (Tian et al., 2017), which retarded the cellular migration without causing acute cytotoxicity (Figure 1E). By exploiting this strong interaction between nanomaterials and proteins, carbon-based nanomaterials have been developed for to inhibit the fibrillation of amyloid-beta peptide or α-synuclein monomer, as well as effectively clearing the mature fibrils by destructively extracting peptide molecules from fibrils (Figure 1F) (Yang et al., 2015; Kim et al., 2019).

## Redox Reaction at the Nano-Bio Interface

Redox reaction at the nano–bio interface is another critical factor that regulates the functions and toxicities of nanomaterials. Nanomaterials interact with these redox-related chemical species by generating and/or scavenging reactive oxygen species (ROS), which influences the fate of cells *in vivo*. Therefore, research on the interaction between nanomaterials and ROS not only help us to understand the mechanism of nanomaterial toxicity, but also broadens the applications of nanomaterials in medicine.

**Figure 2:**
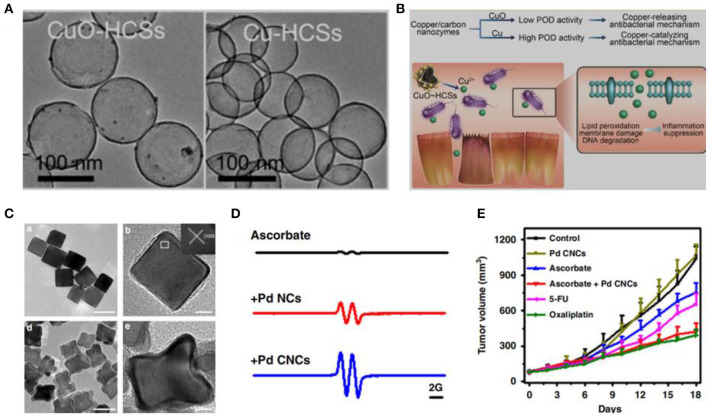
Redox reaction at the nano-bio interface. **(A)** TEM images of copper/carbon nanozymes. **(B)** Tuning catalytic activity by the copper state for antibacterial therapy. **(C)** TEM images of Pd nanocrystals. (Xi et al., 2019) (with permission of American Chemical Society). **(D)** Oxidation of ascorbate catalyzed by Pd nanocrystals. **(E)** Tumor growth curves from HCT116 tumor with ascorbate or Pd nanocrystals (Chong et al., 2018) (with permission of Springer Nature).

Numerous studies have demonstrated that many types of engineered nanomaterials are capable of accomplishing natural enzyme-like catalytic performance. For instance, iron oxide nanoparticles (Fe_3_O_4_) (Chen et al., 2012), graphene quantum dots (GQDs) (Sun et al., 2014), and Au nanoparticles (Wang et al., 2017) have oxidase- and/or peroxidase (POD)-like activities. In addition, we have investigated GO, GQDs, silver (Ag) nanoparticles, Pd nanoparticles, and Pd@Ir nanoparticles that have ROS-generating abilities (Chong et al., 2016, 2017; Ge et al., 2016; Fang et al., 2018; Cai et al., 2019; Tian et al., 2019). The ROS-generating abilities of these nanomaterials differ in sizes, shapes, and facets. For instance, Gao et al. have found that the POD-like activity of cooper nanoparticles is state-dependent (Figures 2A,B) (Xi et al., 2019). Furthermore, we have found that Pd nanoparticles with oxidase-like activity can catalyze the oxidation of ascorbate and generate H_2_O_2_. Pd nanoparticles enclosed by high-index facets remarkably amplify the oxidation of ascorbate, which is selective against cancer cells (Figures 2C–E) (Chong et al., 2018).

The scavenging of overexpressed ROS in pathological sites has been employed as a general therapeutic approach to pathological abnormalities, such as, Alzheimer's disease (Kwon et al., 2016), hepatitis (Zhang et al., 2016), and radiation damage (Cheng et al., 2018). Therefore, nanomaterials with catalase (CAT)- and/or superoxide dismutase (SOD)-like activities have been studied as therapeutic agents in ROS-related diseases. Metal-based nanomaterials, such as, cerium oxide (CeO_2_), manganese oxide (Mn_3_O_4_), Pd, and Pt, have attracted extensive attention due to their excellent enzyme-like activities (Chen et al., 2016; Kwon et al., 2016; Singh et al., 2017). For instance, custom-made CeO_2_ nanoparticles possessed SOD-like activity and can catalyze the reaction of ${\text{O}}_{2}^{-}$ to generate O_2_. These nanoparticles showed an excellent ability to protect neuronal cells from oxidative damage (Zeng et al., 2018). It is expected that these nanomaterials with a strong ability to scavenge ROS could be developed as a promising therapeutic agent for oxidative stress-related diseases.

## Conclusions and Perspectives

Research on the nano–bio interfaces of engineered nanomaterials is an important issue in the development of nanomedicine. This is because nano–bio interfaces are related to the intelligent design of safe and effective nanomedicine, drug delivery, pathological site targeting, metabolism, and biocompatibility. In this review, we summarized recent advances in nano–bio interactions of nanomaterials from the perspective of corona and redox reactions. With these advances, the future use of nanomaterials in biomedicine will hold great promise, especially in ROS-related diseases. Nevertheless, the research of nano–bio interfaces still has many challenges: (1) A full understanding of the catalytic mechanisms of nanomaterials toward redox species is still lacking. (2) Regulation strategy on the catalytic activity of nanomaterials needs to be developed for their effective application as smarter therapeutic and diagnostic modalities. (3) Research on nano–bio interactions needs to consider the complex environment *in vivo*. (4) More attention should be paid to theoretical simulation in order to accurately and deeply investigate the nano–bio interactions. Thus, more efforts should be made in the research of nano–bio interactions.

## Author Contributions

The manuscript was prepared by XT and YC, who contributed equally. CG conceived the review and contributed to the improvement of the manuscript.

## Conflict of Interest

The handling Editor declared a past co-authorship with one of the authors CG. The remaining authors declare that the research was conducted in the absence of any commercial or financial relationships that could be construed as a potential conflict of interest.
