# Supplementary material for: Understanding the Nano–Bio Interactions and the Corresponding Biological Responses
Source: Front Chem. 2020 Jun 10;8:446. doi: 10.3389/fchem.2020.00446 (PMC7298559; doi:10.3389/fchem.2020.00446)
Supplement: Supplementary file 1 [file Data_Sheet_1.DOCX]

**Supporting Information**

**for**

**Understanding the Nano−Bio Interactions and the Corresponding Biological Responses**

***Xin Tian^#^, Yu Chong^#^, and Cuicui Ge^*^***

*State Key Laboratory of Radiation Medicine and Protection, School of Radiation Medicine and Protection, School for Radiological and Interdisciplinary Sciences (RAD-X), Collaborative Innovation Center of Radiation Medicine of Jiangsu Higher Education Institutions, Soochow University, Suzhou 215123, China*

***Correspondence:** Cuicui Ge (ccge@suda.edu.cn)

^#^ Each author contributed equally.

**Scheme S1. The interaction between nanomaterials and bio-environment at nano-bio interfaces, including physical adsorption performance and chemical redox.**
